# Custom‐Made Mouthpiece‐Assisted Oral Cryotherapy During Hematopoietic Stem Cell Transplantation in a Patient With Hemimaxillectomy‐Related Oroantral Communication: A Case Report

**DOI:** 10.1002/ccr3.73156

**Published:** 2026-07-17

**Authors:** Kumiko Matsuzaki, Fuminobu Miyazaki, Yasuji Motoyama, Seiko Takeda, Takuo Kuboki, Yoshihiko Soga

**Affiliations:** ^1^ Division of Hospital Dentistry Okayama University Hospital Okayama Japan; ^2^ Dental Laboratory Division Okayama University Hospital Okayama Japan; ^3^ Department of Oral and Maxillofacial Reconstructive Surgery Okayama University Hospital Okayama Japan; ^4^ Department of Oral Rehabilitation and Regenerative Medicine Okayama University Graduate School of Medicine, Dentistry and Pharmaceutical Sciences Okayama Japan

**Keywords:** hematopoietic stem cell transplantation, hemimaxillectomy, mouthpiece, oral cryotherapy, oral mucositis

## Abstract

A custom‐made mouthpiece enabled safe oral cryotherapy in a patient with extensive oroantral communication after hemimaxillectomy and may also be applicable to other patients with communication between the oral cavity and the maxillary sinus or nasal cavity.

## 1 Introduction

Hematopoietic stem cell transplantation (HSCT) is a procedure in which hematopoietic stem cells are administered to reconstitute or replace the hematopoietic system. HSCT is used across a wide range of indications, particularly hematologic diseases such as acute leukemia, malignant lymphoma, multiple myeloma, myelodysplastic syndromes, and bone marrow failure syndromes. It is broadly classified into autologous HSCT, in which the patient's own hematopoietic stem cells are used, and allogeneic HSCT, in which hematopoietic stem cells are obtained from a donor [1]. In Japan, more than 5300 hematopoietic cell transplants, including both autologous and allogeneic procedures, were reported in 2024. Although the number of patients with extensive communication between the oral cavity and the maxillary sinus or nasal cavity is expected to be limited, this anatomical situation can make standard oral cryotherapy difficult or potentially unsafe in selected patients [2].

Oral mucositis is one of the most common and clinically significant adverse events associated with HSCT, with reported incidences ranging from 47% to 100% [3, 4]. Clinically, oral mucositis can cause painful erythematous or ulcerative lesions, difficulty eating and swallowing, impaired oral hygiene, and reduced quality of life. In immunocompromised patients, disruption of the oral mucosal barrier may also increase susceptibility to local and systemic infections [5, 6]. Melphalan is a well‐established cause of oral mucositis, and oral mucositis is regarded as one of its dose‐limiting toxicities [7]. Because melphalan is rapidly cleared from plasma, oral cryotherapy is considered a rational preventive intervention to reduce mucosal exposure to circulating melphalan [8, 9]. During oral cryotherapy, patients typically hold ice chips in the mouth shortly before, during, and after melphalan infusion, often for a total duration of approximately 1 h. This cooling induces vasoconstriction in the oral mucosa and is thought to reduce mucosal exposure to circulating melphalan. Previous clinical studies in allogeneic HSCT recipients and a MASCC/ISOO systematic review have shown that oral cryotherapy can reduce the incidence and severity of melphalan‐induced oral mucositis [10, 11, 12]. The MASCC/ISOO clinical practice guidelines recommend oral cryotherapy for patients undergoing autologous HSCT when the conditioning regimen includes high‐dose melphalan [6].

In most patients, oral cryotherapy is simple and noninvasive. However, it may be difficult to perform in patients with large maxillary defects and extensive communication between the oral cavity and maxillary sinus. In such cases, ice fragments may enter the maxillary sinus and cold water may leak into the nasal cavity. In addition, use of an obturator alone may result in inadequate cooling of the mucosa covered by the prosthesis. Here, we report a case in which a custom‐made mouthpiece enabled oral cryotherapy to be performed safely in a patient with hemimaxillectomy‐related oroantral communication undergoing allogeneic HSCT.

## 2 Case History/Examination

A 29‐year‐old woman was referred to our department for oral management before allogeneic HSCT. She had relapsed acute myeloid leukemia and had been transferred to our hospital for a third transplantation. Myeloablative conditioning with fludarabine, busulfan, and melphalan was planned. She was first seen 17 days before the start of the conditioning regimen. She had not previously received melphalan‐containing conditioning or oral cryotherapy before the present transplantation. Detailed information on the severity of oral mucositis during prior treatments was not available in the records at our institution.

Her medical history was notable for left maxillary actinomycosis, for which she had previously undergone left hemimaxillectomy and fabrication of a maxillary obturator. Oral examination revealed a wide defect extending from the left maxillary central incisor region to the left maxillary molar region, with extensive oroantral communication on the left side (Figure 1A). A maxillary prosthesis was in place and showed good adaptation (Figure 1B). The obturator had an open‐top design and was inserted and removed through the defect (Figure 1C). She also had limited mouth opening. Panoramic radiography confirmed resection of the left maxilla (Figure 2).

**FIGURE 1:**
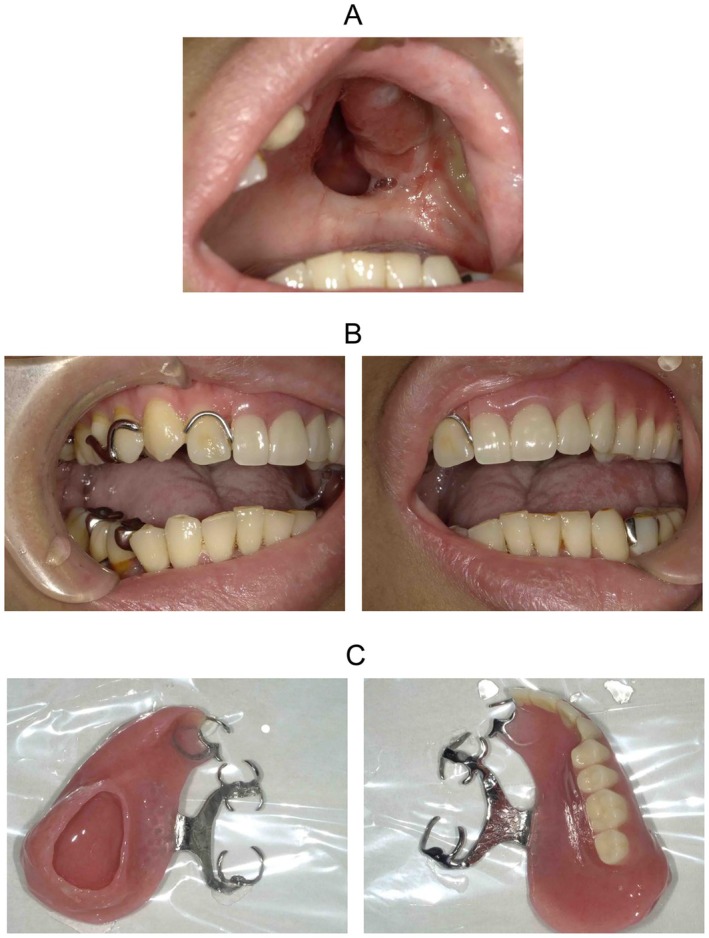
Pretreatment intraoral findings and maxillary prosthesis. (A) Intraoral view showing a defect of the left maxilla. (B) Intraoral view with the maxillary obturator in place. (C) The obturator had an open‐top design and showed excellent adaptation.

**FIGURE 2:**
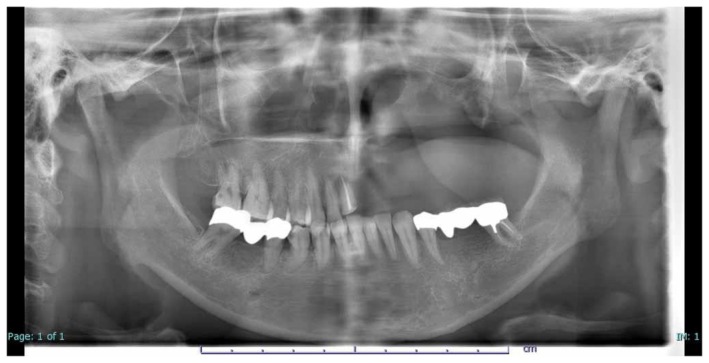
Pretreatment panoramic radiograph. Panoramic radiograph showing absence of the left maxillary teeth and maxillary bone. The right maxillary central incisor had undergone root canal treatment and remained as a residual root.

Because the conditioning regimen included melphalan, oral cryotherapy was considered indicated. However, cryotherapy without the prosthesis would have allowed intrusion of ice into the maxillary sinus and leakage of cold water into the nasal cavity. Conversely, cryotherapy with the obturator in place was expected to result in insufficient cooling of the mucosa covered by the denture base. We therefore planned to fabricate an additional device that would close the defect while allowing oral cryotherapy.

Because the patient had refractory acute myeloid leukemia and was not in remission, she received additional chemotherapy with CPX‐351 after her initial dental visit. Her neutrophil count then decreased to below 500/μL, and she was no longer able to leave the clean room. Device planning and treatment were therefore performed at the bedside in the protective environment.

The major clinical problem in this case was not diagnostic uncertainty but the safe implementation of oral cryotherapy in the presence of a known maxillary defect and extensive oroantral communication. The anatomical condition was confirmed by the patient's surgical history, oral examination, and panoramic radiography. Before transplantation, no active odontogenic infection or ulcerative oral lesion requiring treatment was observed. During follow‐up after transplantation, the oral mucosa was monitored for ulcerative oral mucositis, traumatic injury related to the device, and signs of local infection.

## 3 Methods

As an initial approach, we attempted to fabricate a device based on the shape of the existing obturator. The prosthesis was scanned, and a hollow device designed to obtain retention from the undercut of the defect was created using design software and produced with a 3D printer using a biocompatible clear resin. The internal space was filled with distilled water, sealed, and frozen (Figure 3). However, at trial fitting, retention proved insufficient because of the weight of the ice, and the device was judged to be clinically impractical.

**FIGURE 3:**
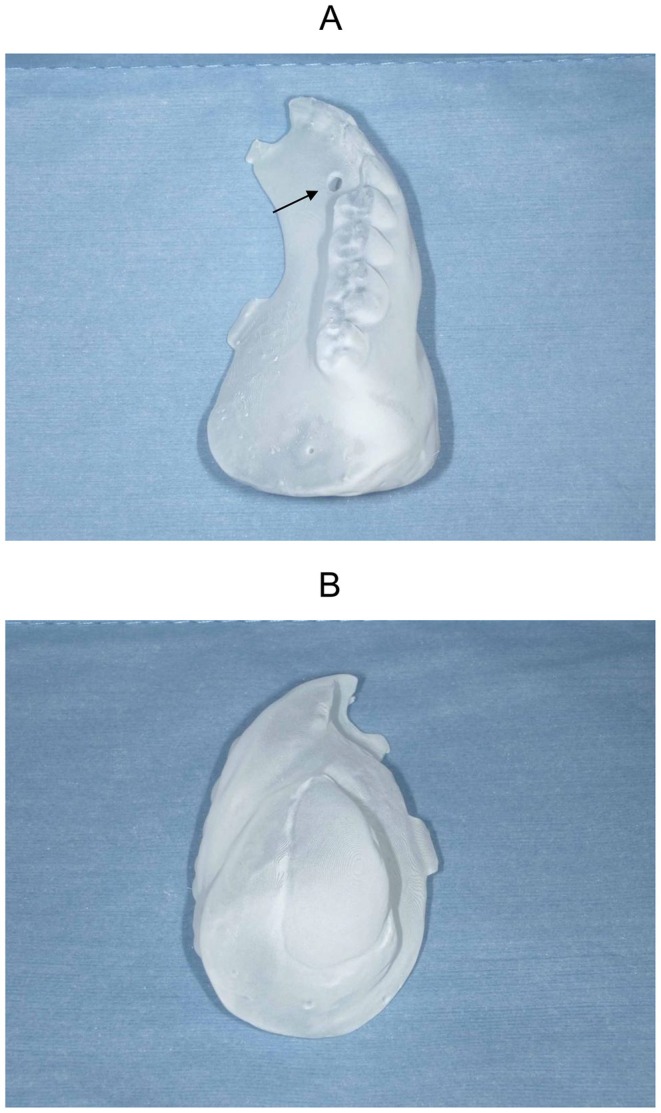
Prototype device for oral cryotherapy. A trial device was fabricated to close the maxillary defect. (A) Distilled water was injected through the opening (arrow), sealed, and frozen. (B) The device had a hollow structure to create a space for water.

We therefore proceeded with a second approach. A dental impression was obtained with the maxillary prosthesis in place using an alginate impression material and a stock tray, allowing the prosthesis and surrounding oral structures to be recorded together. To minimize the risk of impression material entering the maxillary sinus, no material was applied to the tissue surface of the prosthesis. Because the obturator had an open‐top design, the open portion was filled with silicone impression material, and other undercuts were relieved with paraffin wax before pouring dental stone. This allowed fabrication of a working model reproducing the contour of the mucosa beneath the prosthesis as well as the remaining palate and dentition. Using this model, a 1.0‐mm hard polyester sheet was pressure‐formed to create a mouthpiece covering the maxillary defect, the remaining palate, and the right maxillary dentition, with retention also obtained from the teeth (Figure 4). At the fitting appointment, the device showed good adaptation, no pain during placement, and no leakage of water into the nasal cavity during drinking.

**FIGURE 4:**
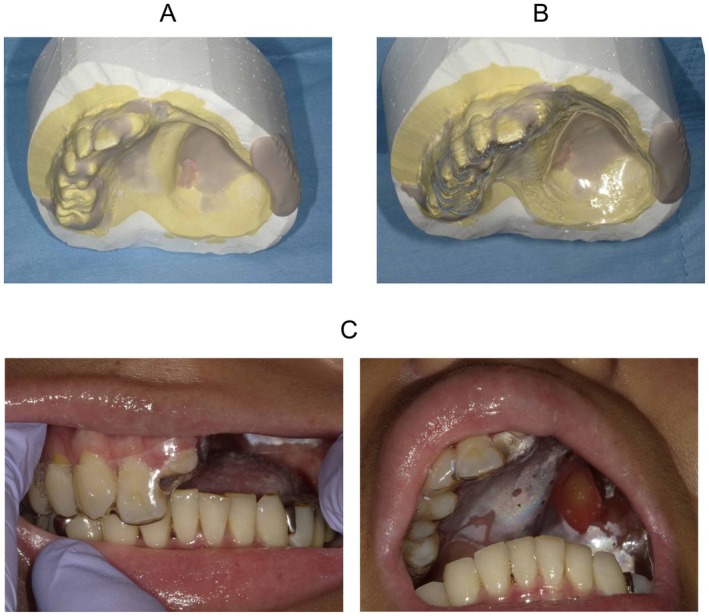
Fabrication and fitting of the custom‐made mouthpiece. A hard polyester sheet was used to fabricate a mouthpiece covering the maxillary defect, the right side of the palate, and the right maxillary dentition. (A) Dental cast obtained using a dental impression with the prosthesis in place. (B) Mouthpiece on the dental cast. (C) Intraoral views of the mouthpiece. The device showed good adaptation. Drinking water confirmed no leakage into the nasal cavity, and oral cryotherapy was performed.

Myeloablative conditioning was then initiated with fludarabine at 30 mg/m^2^/day for 6 days, busulfan at 3.2 mg/kg/day for 4 days, and melphalan at 40 mg/m^2^/day for 2 days. Oral cryotherapy was performed on both days of melphalan administration. The patient began holding ice in her mouth 15 min before melphalan infusion, continued during the approximately 30‐min infusion, and maintained cryotherapy for 30 min after infusion. The total duration was approximately 75 min per session. During both sessions, no ice fragments entered the maxillary sinus, and no cold water leaked into the nasal cavity. The procedure was completed without complications.

## 4 Outcome and Follow‐Up

The custom‐made mouthpiece enabled oral cryotherapy to be performed as planned on both days of melphalan administration. During both sessions, no ice fragments entered the maxillary sinus, no cold water leaked into the nasal cavity, and no procedure‐related complications occurred. These findings indicated that oral cryotherapy could be delivered safely and feasibly in this patient. The patient reported no pain or marked discomfort while wearing the custom‐made mouthpiece and was able to complete oral cryotherapy on both days of melphalan administration. She did not report nasal leakage of cold water during the procedure.

During follow‐up, ulcerative oral mucositis did not develop after transplantation (Figure 5). Engraftment was confirmed on day 15 after transplantation, when the neutrophil count remained above 500/μL for three consecutive days.

**FIGURE 5:**
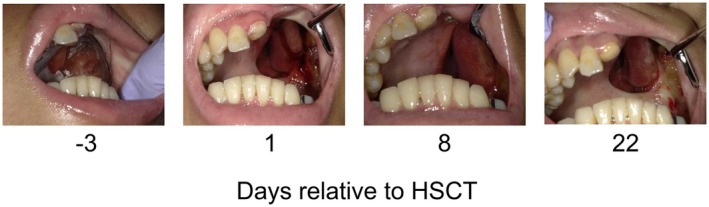
Intraoral changes over time relative to hematopoietic stem cell transplantation (HSCT). The photographs are arranged from left to right according to the day relative to HSCT: Day −3, day 1, day 8, and day 22. On day −3, the mouthpiece for oral cryotherapy was in place immediately before melphalan administration. On day 1, erythema and bleeding marks were observed on the palatal mucosa around the defect and the mucobuccal fold in the left maxillary molar region. On day 8, erythema persisted on the palatal mucosa around the defect. On day 22, residual bleeding marks were observed on the palatal mucosa around the defect.

## 5 Discussion

This case is clinically important because it illustrates a practical method for performing oral cryotherapy in a patient after hemimaxillectomy with extensive oroantral communication. Although oral cryotherapy is recommended in the MASCC/ISOO guidelines for autologous HSCT with high‐dose melphalan [6] and is supported by clinical studies in allogeneic HSCT recipients and a MASCC/ISOO systematic review [10, 11, 12], standard administration with ice chips may not be feasible in patients with major maxillary defects. In the present case, the principal concerns were intrusion of ice into the maxillary sinus, leakage of cold water into the nasal cavity, and insufficient cooling of the mucosa if cryotherapy were performed with the obturator alone.

A key issue in this case was how to obtain a safe and usable device under difficult clinical conditions. Impression taking in patients with maxillary defects requires special caution because impression materials may enter deep undercuts or the maxillary sinus, making removal difficult and potentially traumatic [13]. In our patient, these concerns were amplified by limited mouth opening, bedside management in a clean room, and profound immunocompromise during the transplantation period. For these reasons, we initially sought to avoid impression taking altogether and attempted fabrication of a 3D‐printed device based on the existing prosthesis.

However, the initial 3D‐printed design was not clinically feasible because the device did not provide adequate retention once filled with ice. This unsuccessful attempt was nevertheless informative, because it clarified the mechanical limitations of a defect‐based design in this patient and supported the need for a second, more stable approach. Rather than weakening the case, this step demonstrates the clinical reasoning that led to the final design.

The final mouthpiece was fabricated from a dental impression obtained with the obturator in place. This approach minimized the risk of impression material entering the maxillary sinus while enabling reproduction of the mucosal contour beneath the prosthesis, the remaining palate, and the dentition needed for retention [13]. The resulting pressure‐formed mouthpiece provided a practical balance of coverage, stability, and patient comfort. Importantly, it prevented both intrusion of ice into the defect and leakage of cold water into the nasal cavity while allowing cryotherapy to be completed as planned.

The favorable clinical course in this case should be interpreted cautiously. Ulcerative oral mucositis did not occur after transplantation, but a single case cannot establish the preventive efficacy of the device itself. The main significance of this report is not that the mouthpiece prevented mucositis per se, but that it enabled safe and feasible delivery of a supportive care intervention supported by previous clinical studies and related clinical practice guidelines in a patient for whom standard oral cryotherapy would otherwise have been difficult or potentially unsafe [6, 10, 11].

Although the present case involved a patient after hemimaxillectomy with extensive oroantral communication, this approach may also be applicable to other patients with communication between the oral cavity and the maxillary sinus or nasal cavity, such as those with an oroantral fistula, cleft palate, or posttreatment maxillary defects. However, the optimal design may differ according to the size and shape of the defect, the presence or absence of a prosthesis, and the available dentition. Further accumulation of cases will be needed to clarify the range of indications and technical refinements for this approach.

## 6 Conclusion

This case demonstrates that a custom‐made mouthpiece can enable safe and feasible oral cryotherapy during melphalan‐containing allogeneic HSCT in a patient after hemimaxillectomy with extensive oroantral communication. This approach may be useful for selected patients with communication between the oral cavity and the maxillary sinus or nasal cavity when standard oral cryotherapy is difficult or potentially unsafe.

## Author Contributions

**Fuminobu Miyazaki:** methodology. **Takuo Kuboki:** supervision. **Yasuji Motoyama:** methodology. **Kumiko Matsuzaki:** conceptualization, methodology, data curation, project administration, writing – original draft, writing – review and editing, investigation, formal analysis. **Yoshihiko Soga:** conceptualization, methodology, supervision, writing – original draft, writing – review and editing. **Seiko Takeda:** methodology, investigation.

## Funding

The authors have nothing to report.

## Ethics Statement

According to the Japanese Ethical Guidelines for Medical and Biological Research Involving Human Subjects, this case report is outside the scope of the guidelines, and formal ethics committee approval was therefore not required.

## Consent

Written informed consent was obtained from the patient for publication of this case report and the accompanying images.

## Conflicts of Interest

The authors declare no conflicts of interest.

## Data Availability

Data sharing not applicable to this article as no datasets were generated or analyzed during the current study.
